# Sensitivity, specificity of biochemical markers for early prediction of endothelial dysfunction in atherosclerotic obese subjects

**DOI:** 10.4314/ahs.v22i2.32

**Published:** 2022-06

**Authors:** Khalid O Abulnaja, Kurunthachalam Kannan, Ashgan Mohammed K Al-Manzlawi, Taha A Kumosani, Mohamed Qari, Said S Moselhy

**Affiliations:** 1 Biochemistry Department, Faculty of Science, King Abdulaziz University, Jeddah, Saudi Arabia; 2 Experimental Biochemistry Unit, King Fahd Medical Research Center, KAU; 3 Department of Pediatric, New York, University School of medicine, New York, NY 10016, USA; 4 Production of Bio-products for Industrial Applications Research Group, KAU; 5 Department of Hematology, Faculty of Medical Science, King Abdulaziz University, Jeddah; 6 Biochemistry Department, Faculty of Science, Ain Shams University, Cairo, Egypt

**Keywords:** Obesity, atherosclerosis, endothelial dysfunction

## Abstract

**Background:**

The obesity increased incidence of diabetes, hypertension and atherosclerosis and rate of morbidity and mortality. The main cause of atherosclerosis is endothelial dysfunction and formation of foam cells and macrophage that lead to unfavorable complications. This study evaluated specific biomarkers for endothelial dysfunction as sensitive indices for early predication of atherosclerosis in obese subjects.

**Study Design:**

One hundred fifty male age and sex matching were included in the current study divided into three groups according to body mass index (BMI): Control (BMI ≤ 22), obese (BMI> 28) and obese with atherosclerosis (BMI> 28). Fasting serum was subjected for determination of adhesion molecules, sICAM-1, sVCAM-1, E-selectin, oxo-LDL and 8-iso-PGF2α by ELISA technique.

**Results:**

Data obtained showed that, a significant elevation of serum inflammatory markers CRP, IL-6 and TNF-α and adhesion molecules sICAM-1 (p<0.001) with sensitivity 96%, sVCAM-1 (p <0.01) with sensitivity 92%, E-selectin (p<0.001) with sensitivity 94%, oxo-LDL (p <0.05) and 8-iso-PGF2α (p < 0.001) with sensitivity 97% in obese with atherosclerosis compared with obese and control.

**Conclusion:**

The levels of serum adhesion molecules contributed in the pathogenesis of endothelial dysfunction can be used as sensitive biomarkers for early prediction of atherosclerosis in obese subjects.

## Introduction

Obesity is considered the most public health problem worldwide. It increases the mortality and the prevalence of cardiovascular diseases, diabetes, and colon cancer1. However, public health intervention programs have had limited success in tackling the rising prevalence of obesity. It is linked with development of different diseases as metabolic syndrome, type II diabetes, hypertension and CVD. Obesity is associated with greater arterial stiffness^1^.

Aterosclerosis is a chronic disease start with accumulation of lipid foam cells in the intimal layer of the artery due to oxidation of LDL-c with peroxiyradicals. This accumulation is the main cause of pathogenesis followed by inflammation in the walls of the major arteries lead to progress to fibroatheromas which are fibrous in nature^2^.

It was reported that, there was an association between C-reactive protein (CRP) and development of metabolic syndrome. Elevated serum high-sensitivity CRP (hs-CRP) level are associated with increased risk of incident cardiovascular events among individuals as having the metabolic syndrome^3^.

The events that contribute in pathogenesis of early atherosclerosis, when LDL-c oxidized in presence of free radical, it is converted to ox-LDL, it can induce activation of endothelial cell, release of inflammatorysubstances and enhance the expression of adhesion molecules, all these events with macrophages increase plaque formation and atherosclerosis^4^. Previous study reported that TNF-α is overexpressed in the adipose tissues of obese and is positively correlated with insulin resistance. The TNF-alpha level was increased in T2D as impaired glucose tolerance. Diabetic patients showed elevation of CRP, IL-6, and TNF- α^5^.

Interleukin 6 (IL-6) is one of cytokines that mediate in acute phase reactions, inflammation, and cancer progression. IL-6 regulates energy homeostasis and capable of inhibiting LPL and control energy intake . It contributes to chronic inflammation in conditions such as obesity, insulin resistance, inflammatory^6^.

Soluble intercellular adhesion molecule-1 (sICAM-1) play an important role in endothelial activation and inflammation. Cellular adhesion molecules mediate the migration of mast cells from the bloodstream to initiate the progression of atherosclerotic plaque^7^. Previous studies reported that, high levels of circulating cell adhesion molecules such as vascular cell adhesion molecule-1 (VCAM-1), and endothelial leukocyte adhesion molecule-1 (E-selectin) are a risk factors for pathogenesis of atherosclerosis^5^. The prostaglandin-like substance (F2-IsoPs) was produced from peroxidation of arachidonic acid by free radicals is implicated in oxidative damage and CVD^6^.

The F2-IsoP is a good indicator of lipid peroxidation and can be used as a significant biomarker of oxidative stress. The 8-Iso-PGF2α is formed in various tissues and can be found in plasma and urine in. Their role in the regulation of normal physiological conditions has yet to be elucidated^6^.

The hypothesis was identification the link between inflammation, metabolic syndrome and endothelial dysfunction

The rational of this study is to validate the adhesion molecules as sensitive biomarkers for diagnosis and prognosis. This study evaluated the sensitivity and specificity of adhesion molecules as a good biomarkers for early predication of endothelial dysfunctions and a major risk in developing atherosclerosis in obese subjects.

## Subjects and Methods

### Study design

In this study, patients with suffered from chest burn of cardiac symptoms hospitalized in the Cardiology Unit, King Abdulaziz Hospital, KAU, Saudi Arabia. The study was carried out from the period Juan 2019 to December 2020. They diagnosed by ECG and ECHO according to the guidelines of the European Association for the Heart Disease. The history of the patients including age, lifestyle, smoking, drug abuse, family history of cardiac diseases, past infection by virus.

### Participant and intervention

One hundred fifty male, age rang (35–65 years) were included in this study. They sorted into 3 groups. Group I: Normal non-obese (BMI<25) .Group II: Obese subjects with no history of CVD (BMI>28). Group III: obese patients (BMI>28) suffering from atherosclerosis with arteries blocked more than 60%. Exclusion criteria include, kidney disease, diabetes and hypertension. Angiography examination for detection the percent of blockage of arteries. All subjects attending King Abdulaziz University Hospital, Internal medicine and Cardiology Unit, Jeddah, Saudi Arabia. All subjects gave informed consent before study and the approval of study according to ethical committee of the king Abdulaziz University.

Measurement of body temperature, weight, pathological changes by using X-ray and blood gases are examples of criteria that have been used successfully to implement humane endpoints

### Methods

Fasting blood samples were collected in plain tubes from all, centrifuged at 3,000×g for 20 minute. Lipid profile (t. cholesterol, triglyceride, HDL-c and LDL-c) were determined by using BIORAD kits, England. Inflammatory markers.

### Assay of CRP

Serum CRP was determined by turbidimetric method according to the method of Particle enhanced immune-turbidimetricassay. Human CRP agglutinates with latex particles coated with monoclonal anti-CRP antibodies^7^.

### Assay of TNF- α

This kit from Biorad (R&D) Systems, Catalog # PDTA00C). The assay is based on the quantitative sandwich enzyme immunoassay technique. A monoclonal antibody specific for TNF-α has been pre-coated onto a microplate^8^.

### Assay of IL-6

This kit used from Biorad (R&D Systems, Catalog # PD6050). This assay employs the quantitative sandwich enzyme immunoassay technique. A monoclonal antibody specific for IL-6 has been pre-coated onto a microplate^9^.

### Assay of serum soluble intercellular adhesion molecule 1 (s ICAM-1) / CD54

This kit used from BIorad (R&D System, Catalog # PDCD540. A monoclonal antibody specific for s ICAM-1 has been pre-coated onto a microplate.

### Assay of serum soluble vascular cell adhesion molecule-1 (s VCAM-1)

This kit used from Biorad (R&D Systems, Catalog # PDVC00). A monoclonal antibody specific for s VCAM-1 has been pre-coated onto a microplate^10^.

### Assay of serum soluble E-Selectin (sE-Selectin)/CD62E

This kit used from (R&D Systems, Catalog # PDSLE00). A monoclonal antibody specific for s E-Selectin has been pre-coated onto a microplate^11^.

### Determination of serum oxidized LDL (oxo-LDL)

This kit from Biorad system Catalog Numbers STA-369) .The oxidized LDL ELISA kit is an enzyme immunoassay developed for the detection and quantitation of human oxLDL in plasma, serum or other biological fluid sample. The kit contains a copper oxidized LDL satandard and has a detection sensitivity limit of < 50 ng/ml^12^.

### Assay of serum 8-iso-prostaglandin F2α

This kit used from Biorad, Catalog Numbers STA-337). A competitive enzyme-linked immunoassay (ELISA) for determining levels of 8-iso-PGF2α in a variety of biological samples such as plasma, serum, urine, or tissue extracts^13^.

### Statistical analysis

All statistical analysis was performed using SPSS statistical software package (SPSS for windows, version 17, SPSS Inc, and USA). One way analysis of variance (ANOVA) was carried out to test the significance of difference between groups mean value for each parameter. Correlation coefficients (r) for pairs of variables were determined by Pearson's method to test the strength of association between any two variables in the same group. Differences were statistically significant at P ≤ 0.05 and highly significant at P ≤ 0.01.

## Results

The sample size of current study included 150 subjects (80 female/70 male), mean age (38 ±7.3). Demographic analysisweight, length and bodymass index (BMI) were mentioned in table 1

**Table 1:** Demographic analysis of the studied Groups (mean ±SD)

Groups	Normal control n=50	Obese n=50	Obese with atherosclerosis n=50	*p-value*
Gender (M:F)	30/20	21/29	16/34	< 0.001
Weight (kg)	69.3 ± 10.8	113.2 ± 10.4	102.2 ± 8.6	< 0.001
Height (cm)	172.5 ± 10.63	171.8 ± 4.0	167.5 ± 7.49	< 0.001
BMI	24.4 ± 2.25	30.2 ± 2.63	31.6 ± 3.43	< 0.001

BMI= body mass index

*P*-value ≤ 0.05 was used as significance.

**Fig 1:**
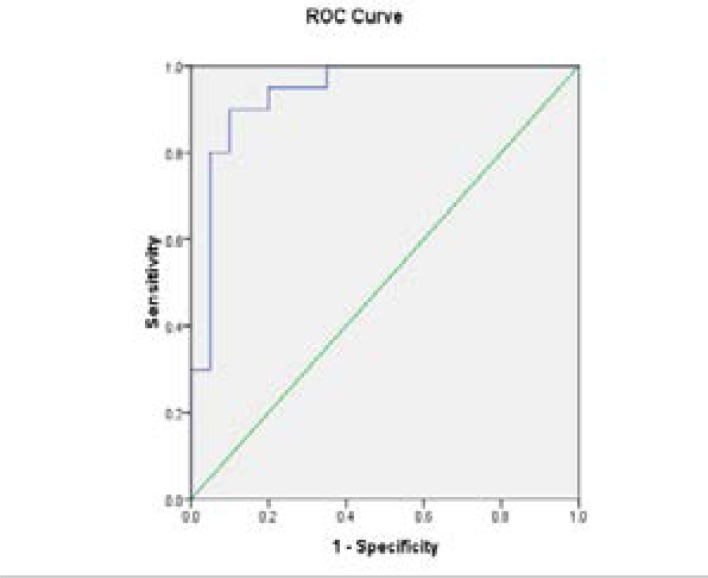
The ROC of E selectin of Obese Vs control

**Fig 2:**
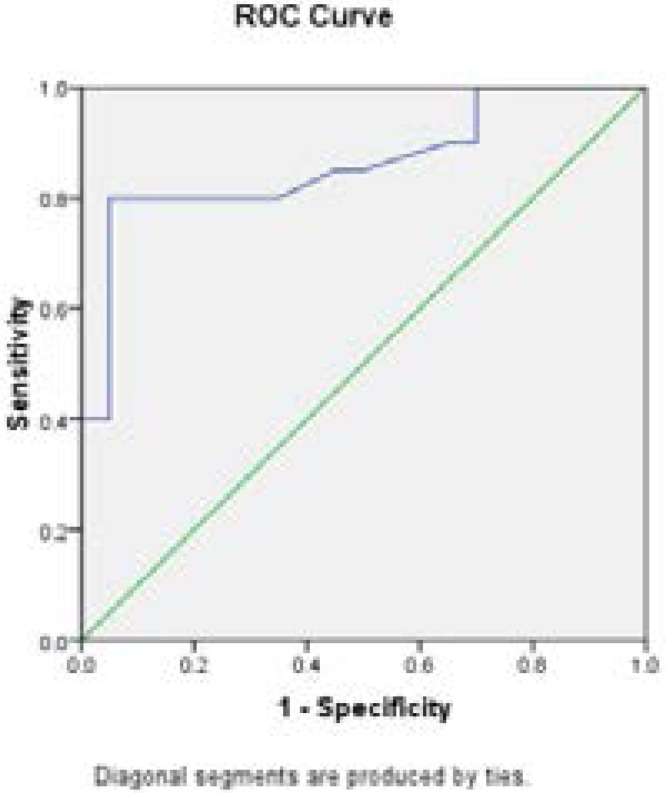
The ROC of E selectin of atherosclerosis Vs Control

Lipid profile in table (2) showed a significant elevation of total cholesterol, triglycerides and LDL-c in obese (2 times) and obese with atherosclerosis (3.5 times) compared with control (p<0.001). However, it is higher in obese with atherosclerosis than obese (p<0.001). The level of HDL-c was decreased in obese and obese with atherosclerosis compared with control (p<0.001). Data obtained in table (3) showed that, there was a significant elevation in the levels of CRP, IL-6 and TNF-α in obese with atherosclerosis patients and obese subjects compared with control group (p < 0.001). In addition, the increases in obese with atherosclerosis was about 5 times than obese. A significant elevation in the level of ICAM-1 in obese and obese with atherosclerosis compared with control group (p < 0.001). However, the level of VCAM-1 was significantly elevated in obese and obese with atherosclerosis compared with control group (p < 0.001) .The mean E-selectin level was significantly higher in obese compared to control (p < 0.001). In addition, there was a significant increase in the mean level of oxo-LDL in obese and obese with atherosclerosis compared to control (p <0.001). Moreover, the level of 8-iso-PGF2α was significantly elevated in obese and obese with atherosclerosis as compared to control (p < 0.001). ANOVA analysis revealed a significant differences between mean value of the studied groups for each of CRP, IL-6, TNF-α, ICAM-1, VCAM-1, E-selectin , oxo-LDL and 8-iso-PGF2α (p= 0.001). The correlation study using Pearson test showed a significant correlation between adhesion molecules with obese and atherosclerosis (table 4). Receiver operating curve (ROC) (table 5, fig 1–2) showed that, the sICAM showed an area under curve (AUC) of 0.93 with (sensitivity 96 % and specificity, 93%). In addition, 8-iso-PGF2α promising in early prediction profile, showing an AUC of 0.9 with (sensitivity, 97.0%; specificity, 95.5%). The sVCAM-1 (p<0.01) showed sensitivity 92%, E-selectin (p<0.001) with sensitivity 94%, oxo-LDL (p<0.05) in obese with atherosclerosis compared with obese and control.

**Table 2:** Lipid profile in all studied groups (Mean ± SD)

Groups	Control (n=50)	Obese (n=50)	Obese and athero. (n=50)
Parameters			
T-Chol (mg/dl)	135.4 ± 14.3	199.1 ± 39.2^a^	235.5 ± 24.6^a.b^
Triglyceride (mg/dl)	80.1 ± 21.2	130.6 ± 22.3 ^a^	141 ± 21.1 ^a.b^
HDL-c (mg/dl)	51.8 ± 7.3	37.5 ± 6.7 ^a^	36.4 ± 4.5 ^a.^
LDL-c (mg/dl)	111.5 ± 12.2	138.6 ± 17.4 ^a^	148.5 ± 17.6 ^a^

n=number of subjects. (a): p <0.05 versu control. (b);p <0.05 versus obese.

**Table 3:** Biomarkers of endothelial dysfunction in different groups (Mean ±SD

Groups	Control (n=50)	Obese (n=50)	Obese with athero (n=50)
Parameters			
CRP(mg/l)	5.19±0.92	17.79±13.92 ^a^	68.79±9.92 ^a.b^
IL-6 (pg/ml)	12.58±0.96	31.05±2.30 ^a^	92.56±13.76 ^a.b^
TNF-α (pg/ml)	5.13±0.59	37.12±3.16 ^a^	128.78±12.1 ^a.b^ 3
ICAM-1 (ng/ml)	11.26±1.22	22.37±3.12 ^a^	45.87±6.16 ^a.b^
VCAM-1 (ng/ml)	18.12±3.37	55.15±5.18 ^a^	95.25±8.38 ^a.b^
E-selectin (ng/ml)	9.18±0.38	32.19±2.01 ^a^	62.79±6.31 ^a.b^
Oxo-LDL (ng/ml)	17.60±0.93	91.35±13.13 ^a^	121.65±16.1 ^a.,b^
8-iso-PGF_2α_ pg/ml	6.00±4.33	22.53±14.04 ^a^	32.113±16.2^a,b^

n=number of subjects. (a): p <0.05 versu control. (b);p <0.05 versus obese.

**Table 4:** Pearson correlation coefficients biochemical marker in control group and obese group

Markers	Obese group r-coefficient	Obese with athero. r-coefficient
ICAM-1	0.001	0.001
VCAM-1	0.05	0.05
E-Selectin	0.01	0.01
Oxo-LDL	0.01	0.01
8-iso-PGF_2α_	0.001	0.001

**Table 5:** Receiver operating curve (ROC) of measured markers as a test for diagnosis of atherosclerosis

Variable	sICAM	sVCAM	E-selectin	8-isoPGE2α
AUC	0.93	0.84	0.87	0.90
Sensitivity (%)	96 %	92%	94%	97%
Specificity (%)	93.1%	87.8%	88%	95.5%

AUC; area under curve

## Discussion

The general obesity is a risk factor for endothelial abnormalities that increased prevalence of CVD^14^. This study investigated the sensitivity and specificity of blood levels of adhesion molecules as a biomarker for early prediction of endothelial dysfunction in obese subjects with atherosclerosis. In the current study, the mean values of CRP and IL-6 were significantly elevated in obese, obese with atherosclerosis compared with control group. This is in agreement with those reported by kern et al^15^, that has shown inflammation plays an important role in the pathogenesis of obesity and cardiovascular disease. Malgorzata et al.^16^, reported that elevated IL-6 concentration is associated with diabetes-related variable which could accelerate progression of micro-vascular complications in DM 1 patients.

Diego et al.^17^ reported that IL-6 appears to be involved in glucose metabolism, insulin resistance, and dyslipidemia in obese children and adolescents with glucose intolerance. In this regard, plasma CRP levels may represent a more appropriate integrated measure of basal IL-6 activity, another potential mechanism that may explain the relationship between inflammation and endothelial dysfunction.

There have been many studies demonstrating an association between CRP and IL-6 and incident diabetes, and several reported this to be independent of adiposity or insulin resistance^18^. Khan et al.^19^ found that C-reactive protein levels were consistently found to be elevated over the increasing grades of obesity in his study, also showed a significant association between cardiovascular risk factors and the likelihood of having elevated CRP in multivariate regression models. It was found that, a significant increase in mean values of oxo-LDL concentration in obese as compared to healthy group. Our results are in agreement with those reported^20^, who found that oxo-LDL level was higher in obese and obese with atherosclerosis versus control. Also, oxo-LDL is associated with plaque and hypertension^21^. Our results are in agreement with those reported^22^, who found the release of free radicals is enhanced from conditions such as ischemia and infection and would be available to oxidize the particle .It was found that, the amount of Ox-LDL appears to be a good marker of atherosclerotic progression. Increased oxidative stress, reflected by elevated levels of oxidized LDL, may contribute in the development of insulin resistance^23^ and therefore be important in the early pathophysiology of type 2 diabetes mellitus. Endothelial dysfunction was diagnosed by evaluation of biomarkers produced from endothelial cells, as adhesion molecules, like sE-selectin, sICAM-1, sVCAM-1. Previous studies reported that, increased levels of adhesion molecules in diabetic patients with endothelial dysfunction^24^. However, this study measure these markers in atherosclerotic subjects.

Results obtained showed that, a significant elevation of adhesions molecules in obese and obese with atherosclerosis compared with control group. The increased in level of circulating sICAM-1, E-selectin and VCAM-1 atherosclerotic obese higher than obese. This is in accordance with results obtained from previous study, the adhesion molecules wereigher 5 times in obese with atherosclerosis and 4 times higher than obese compared with normal control^25^–^27^.

Circulating adhesion molecules sICAM elevated and associated with increased risk of future myocardial infarction in healthy men. Meanwhile, there was a significant elevation in the levels of sICAM and not sVCAM are independently associated with the development of accelerated atherosclerosis among healthy men^28^.

Importantly, weight reduction decreases levels of CRP, IL-6, and TNF-α and lowers sICAM-1, sVCAM-1, and P-selectin .CRP and sICAM-1 as indices of inflammation and vWF and sVCAM-1 as indices of endothelial dysfunction were independently associated with increased CVD mortality, together explaining about 43%of the excess CVD risk^29^.

The ligands of integrins (VCAM-1 and ICAM-1) are expressed in thrombocytes and white blood cells. Their levels in serum elevated in response to inflammatory mediators. Their levels were determined as a biomarker of dysfunction. In addition, they expressed in proatherosclerotic states. It is considered to be a predictor for atherosclerosis and cardiovascular diseases^30^

The 8-iso-PGF2α produced from archidonic acid are associated with vasoconstriction and nephropathy^24^. Data obtained showed that the circulating level of 8-iso-PGF2α was higher in obese and obese with atherosclerosis compared with control. It was twofold higher in obese with atherosclerosis than in obese. The 8-iso-PGF2α play an important role in pro-atherogenic actions^31^ .This is in agreement with the previous study that mentioned that, LDL oxidation and 8-iso-PGF2α are a risk factors for atherosclerotic plaques compared to control^32^.

## Conclusion

The levels of serum adhesion molecules contributed in the pathogenesis of endothelial dysfunction can be used as sensitive biomarkers for early prediction of atherosclerosis in obese subjects.

## Figures and Tables

**Fig 3:**
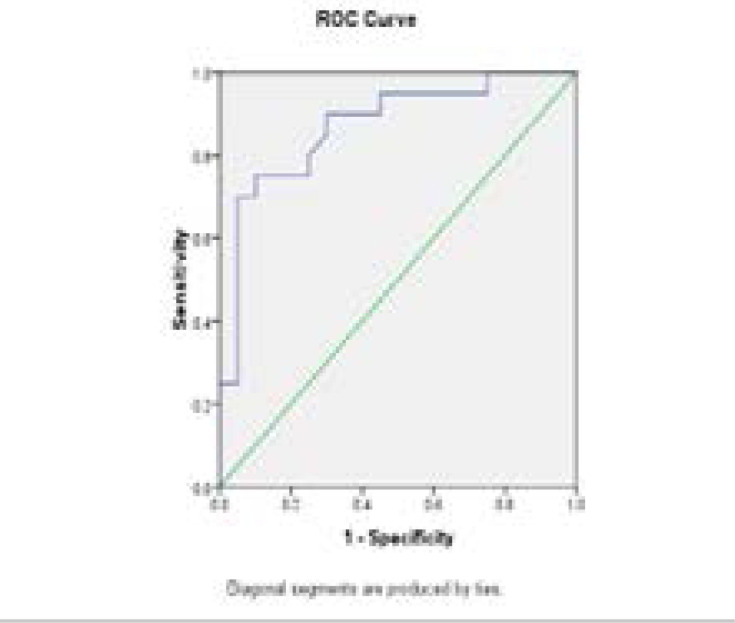
The ROC of E selectin of obese with atherosclerosis Vs Control

## List of abbreviations

Body mass index (BMI); LDL-c oxidized (OX-LDL); Soluble intercellular adhesion molecule-1 (sICAM-1); vascular cell adhesion molecule-1 (VCAM-1); endothelial leukocyte adhesion molecule-1 (E-selectin); prostaglandin-like substance (F2-IsoPs); Receiver operating curve (ROC); C-reactive protein (CRP). area under curve (AUC).
